# Prolonged Diagnostic Pathways and Advanced-Stage Presentation in Early-Onset Colorectal Cancer: A Mexican Cohort Study

**DOI:** 10.7759/cureus.112398

**Published:** 2026-07-10

**Authors:** Andrea Fabiola Hernandez Trejo, Ana Jimena Iberri Jaime, Billy Jimenez Bobadilla

**Affiliations:** 1 Coloproctology, General Hospital of Mexico Dr. Eduardo Liceaga, Mexico City, MEX; 2 Colorectal Surgery, General Hospital of Mexico Dr. Eduardo Liceaga, Mexico City, MEX

**Keywords:** advance stage of disease, colorectal cancer, delay treatment, diagnosis delay, diagnostic delay, early-onset colorectal cancer, stage of diagnosis, time to treatment, tumor staging

## Abstract

Background

Early-onset colorectal cancer (EOCRC) (diagnosed before age 50) is rising worldwide, with the steepest increases in rectal and distal colonic tumors and a disproportionate burden in Latin American populations. Because young patients are diagnosed through symptoms rather than screening, they often present with advanced disease; yet the diagnostic pathway that precedes this presentation - and whether delay shapes stage - remains uncharacterized in Mexico. We aimed to quantify the diagnostic and treatment pathway of Mexican patients with EOCRC and to examine its association with advanced-stage presentation.

Methods

Single-center, retrospective, cross-sectional study of patients younger than 50 years with histopathologically confirmed adenocarcinoma of the colon or rectum managed at a tertiary referral center in Mexico City (January 2016-June 2026). Diagnostic intervals were reconstructed from the medical record using the Aarhus framework. Advanced stages were clinical stages III-IV. Groups were compared (Mann-Whitney U; χ²/Fisher), and multivariable logistic regression identified factors associated with advanced stage; we compared patients with and without computable intervals and performed a sensitivity analysis in those diagnosed from 2019 onward.

Results

Among 226 patients (median age 41 years (IQR 35-45); 91 (40%) aged <40 years; 118 (52%) male), tumors were located in the rectum in 121 (54%). Advanced stages (III-IV) were present in 176/226 (78%). The median total interval from symptom onset to treatment was 243 days (IQR 142-407); 72/108 (67%) exceeded 180 days, and the diagnostic interval exceeded 90 days in 51/141 (36%). Delay intervals did not differ between advanced- and early-stage disease (all p > 0.33), a finding confirmed in a sensitivity analysis restricted to patients diagnosed from 2019 onward. The advanced stage was independently associated with rectal location (adjusted OR 2.97, 95% CI 1.52-5.78; p = 0.001) and higher carcinoembryonic antigen (CEA) at diagnosis (p < 0.001).

Conclusions

Young Mexican patients with EOCRC present overwhelmingly with advanced disease after markedly prolonged diagnostic pathways (median ~8 months). Although delay was not statistically associated with stage, the high burden of advanced presentation and rectal predominance highlights an actionable gap in early recognition and timely referral.

## Introduction

Colorectal cancer (CRC) remains one of the leading causes of cancer-related morbidity and mortality worldwide. According to GLOBOCAN 2022, CRC is the third most commonly diagnosed cancer and the second leading cause of cancer death globally, with an estimated 1.93 million new cases and 904,019 deaths in 2022 [1]. In Mexico, CRC is a growing public-health concern, with 16,082 new cases reported in 2022 [1-2].

Although CRC has traditionally been a disease of older adults, its shift toward younger patients [3-4] has prompted lowering the recommended screening start age from 50 to 45 years [5]. In the United States, 22% of CRCs were diagnosed before age 55 in 2022, double the 11% recorded in 1995, even as incidence in adults aged 20-49 years continues to rise by approximately 3% per year [3,6-7]. By 2024, CRC had become the leading cause of cancer death in men younger than 50 and the second in women of the same age [6]. Rectal tumors in particular have increased by about 1% per year since 2018 and now account for roughly one third of all CRC, with the steepest rises among younger patients and distal cancers [3-4].

Critically, early-onset colorectal cancer (EOCRC) (diagnosed before age 50) is frequently detected late: in recent United States data, approximately three of every four CRCs in adults younger than 50 are diagnosed at an advanced regional or distant stage [3]. Because most young patients fall below the screening age and are identified through symptoms rather than screening, the diagnostic pathway - the time from first symptom to diagnosis and treatment - becomes a central determinant of stage and outcome [8-11]. EOCRC may also exhibit distinct clinicopathological features, including poor differentiation, mucinous or signet-ring histology, a predominance of distal colon and rectal tumors, and higher rates of microsatellite instability [12-15].

This shift is especially relevant to Latin American and Hispanic populations, among whom early-onset incidence is rising rapidly [6]. At our institution, a recent comparison of early- versus late-onset CRC documented a distinct clinical profile in young patients, with a predominance of distal colon and rectal tumors, advanced stage, and adverse histology [16]. However, the diagnostic and treatment pathway itself - how long young patients take to reach diagnosis and treatment, and whether such delay shapes the stage at presentation - has not been characterized in this population.

Because most young patients fall below the screening age and are diagnosed through symptoms rather than screening, the time to diagnosis becomes a central and potentially modifiable determinant of stage and outcome. We therefore set as our primary objective to quantify the diagnostic and treatment pathways of young Mexican patients with EOCRC using the Aarhus framework and to evaluate their association with advanced-stage presentation at a tertiary referral center. As secondary objectives, we aimed to describe the individual Aarhus intervals (patient, diagnostic, treatment, and total) and the proportion of prolonged pathways, and to identify clinicopathological factors independently associated with advanced stage.

## Materials and methods

Study design and setting

We conducted a single-center, retrospective, cross-sectional study at the Department of Coloproctology of the Hospital General de México “Dr. Eduardo Liceaga,” a tertiary referral center in Mexico City, reviewing the records of patients with EOCRC managed between January 2016 and June 2026. The study was reported following the Strengthening the Reporting of Observational Studies in Epidemiology (STROBE) statement [17].

Participants

We included patients younger than 50 years at diagnosis with histopathologically confirmed adenocarcinoma of the colon or rectum. We excluded anal canal carcinomas, confirmed hereditary syndromes (familial adenomatous polyposis (FAP) or Lynch), non-adenocarcinoma histologies, non-incident (recurrent/prevalent) cases, and patients without histopathological confirmation of invasive colorectal adenocarcinoma. To minimize case loss, the cohort was assembled through multi-source ascertainment (a departmental sessioned registry, institutional census, and interconsultation lists, a live CRC-clinic session sheet, preoperative and pathology notes, and a prior departmental EOCRC database); overlap between sources (capture-recapture; ~69 cases shared across sources) supports ascertainment completeness. Patients were de-duplicated by their unique clinical record number.

Data sources and variables

Clinical data were abstracted from the medical record (the institutional electronic health record and departmental clinical notes and oncology session sheets), and the diagnostic timeline was reconstructed from the documented dates. Following the Aarhus framework, we defined the patient interval (symptom onset → first medical contact), the diagnostic interval (first contact → histopathological diagnosis), the treatment interval (diagnosis → first cancer-directed treatment), and the total interval (symptom onset → treatment) [18].

Operational definitions

Following the Aarhus framework [18], symptom onset was taken from the documented history; when recorded only as a duration (“X months of evolution”), it was approximated to the first day of the corresponding month. Initiation of treatment was defined as the first cancer-directed therapy (tumor resection or the start of chemotherapy or radiotherapy); a diverting stoma was not considered treatment. When the diagnosis was established on the surgical specimen, the date of resection served as both the diagnostic and treatment date. Advanced disease was defined as clinical stage III-IV (AJCC/TNM, eighth edition).

Statistical analysis

Continuous variables are reported in medians (IQR) and categorical variables in frequencies (%). Early- (I-II) and advanced-stage (III-IV) disease were compared with the Mann-Whitney U test for continuous variables and the χ² test for categorical variables; the φ coefficient was reported as the effect size for 2 × 2 comparisons. Multivariable logistic regression identified factors associated with advanced stage (odds ratios (OR), 95% CI). To check the consistency of the findings, we compared the characteristics of patients with and without computable intervals and performed a sensitivity analysis restricted to patients diagnosed from 2019 onward. No imputation was performed; each analysis included all patients with available data for the variables involved (complete-case analysis), and the number of evaluable patients is reported for every interval. A two-sided p < 0.05 was significant. Analyses were performed in Python 3.10 (pandas, NumPy, SciPy) (Python Software Foundation, Wilmington, DE).

Ethics

The study was conducted in accordance with the principles of the Declaration of Helsinki. Patient identifiers were dissociated from the analytic dataset (study folio only; the name/record-number key was stored separately).

## Results

**Figure 1:**
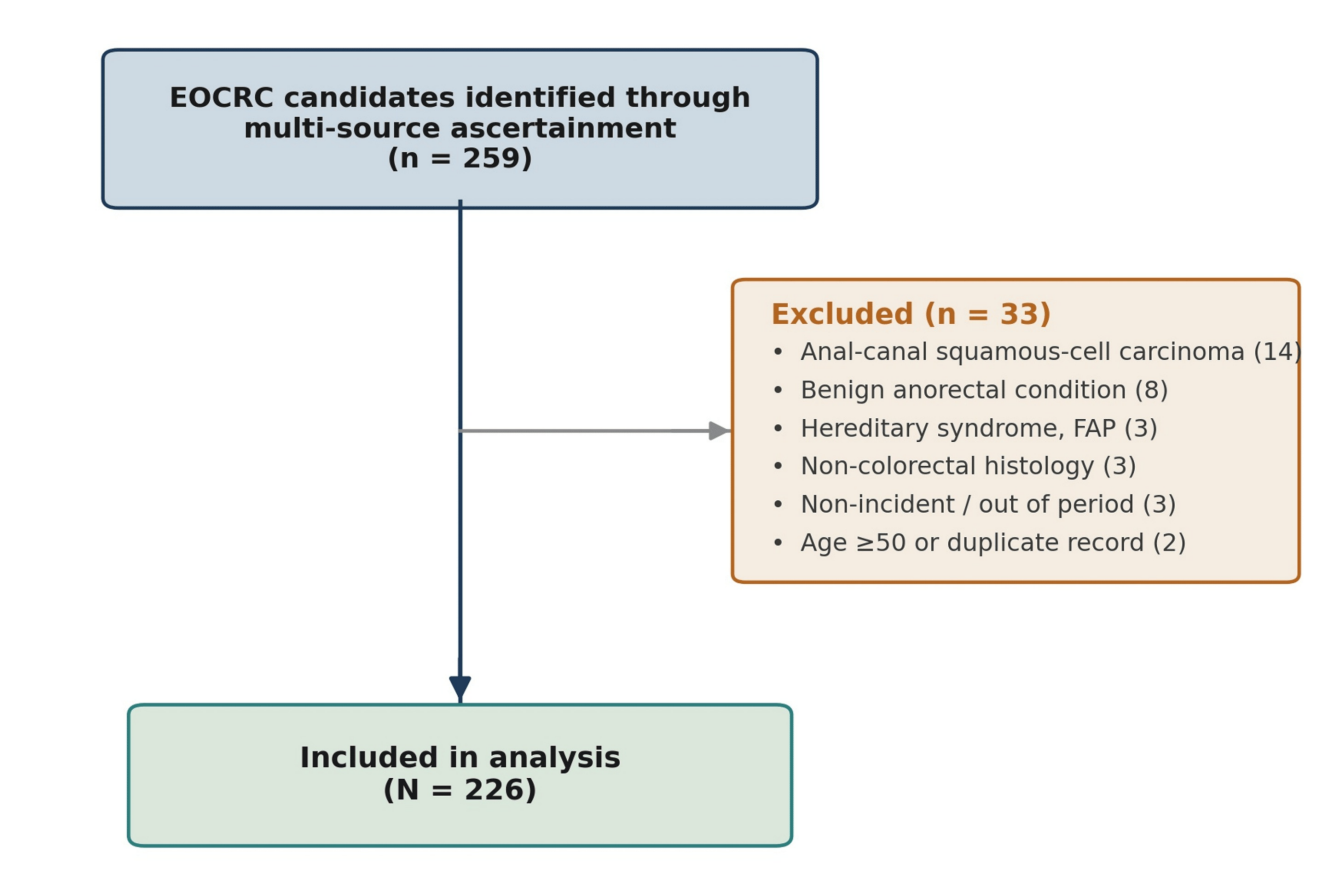
Study flow diagram showing cohort assembly through multi-source ascertainment and reasons for exclusion.

**Figure 2:**
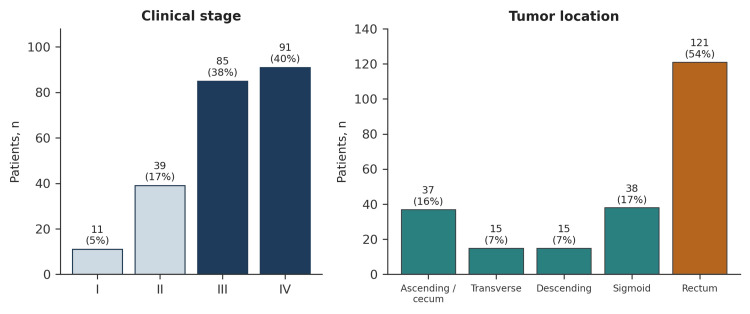
Distribution of clinical stage (left) and tumor location by segment (right); N = 226.

A total of 226 patients met the inclusion criteria (Figure 1; Table 1). Median age was 41 years (IQR 35-45; range 17-49); 91 (40%) were younger than 40 years, and 118 (52%) were male. Tumors were located in the rectum in 121 (54%), the sigmoid colon in 38 (17%), the ascending colon or cecum in 37 (16%), the descending colon in 15 (7%), and the transverse colon in 15 (7%). Clinical stages were III-IV (advanced) in 176/226 (78%). Histology was conventional adenocarcinoma (not otherwise specified (NOS)) in 191 (87%), mucinous in 23 (11%), and signet-ring in five (2%) (Figure 2).

**Table 1:** Baseline characteristics (N = 226). BMI, body mass index; CRC, colorectal cancer; IQR, interquartile range (central 50% of patients, 25th-75th percentiles); NOS, not otherwise specified

Characteristic	Value
Demographic and clinical characteristics	
Age at diagnosis, years - median (IQR)	41 (35-45)
Age <40 years - n (%)	91 (40%)
Male sex - n (%)	118 (52%)
Body mass index, kg/m² - median (IQR) (n = 150)	25.0 (21.5-28.8)
Type 2 diabetes - n/N (%)	18/134 (13%)
Current/former smoking - n/N (%)	64/135 (47%)
Alcohol use - n/N (%)	74/139 (53%)
Family history of CRC - n/N (%)	22/135 (16%)
Tumor location - n (%)	
Ascending colon/cecum	37 (16%)
Transverse colon	15 (7%)
Descending colon	15 (7%)
Sigmoid colon	38 (17%)
Rectum	121 (54%)
Clinical stage - n (%)	
I	11 (5%)
II	39 (17%)
III	85 (38%)
IV	91 (40%)
Histology - n (%)	
Conventional adenocarcinoma (NOS)	191 (87%)
Mucinous	23 (11%)
Signet-ring cell	5 (2%)

**Figure 3:**
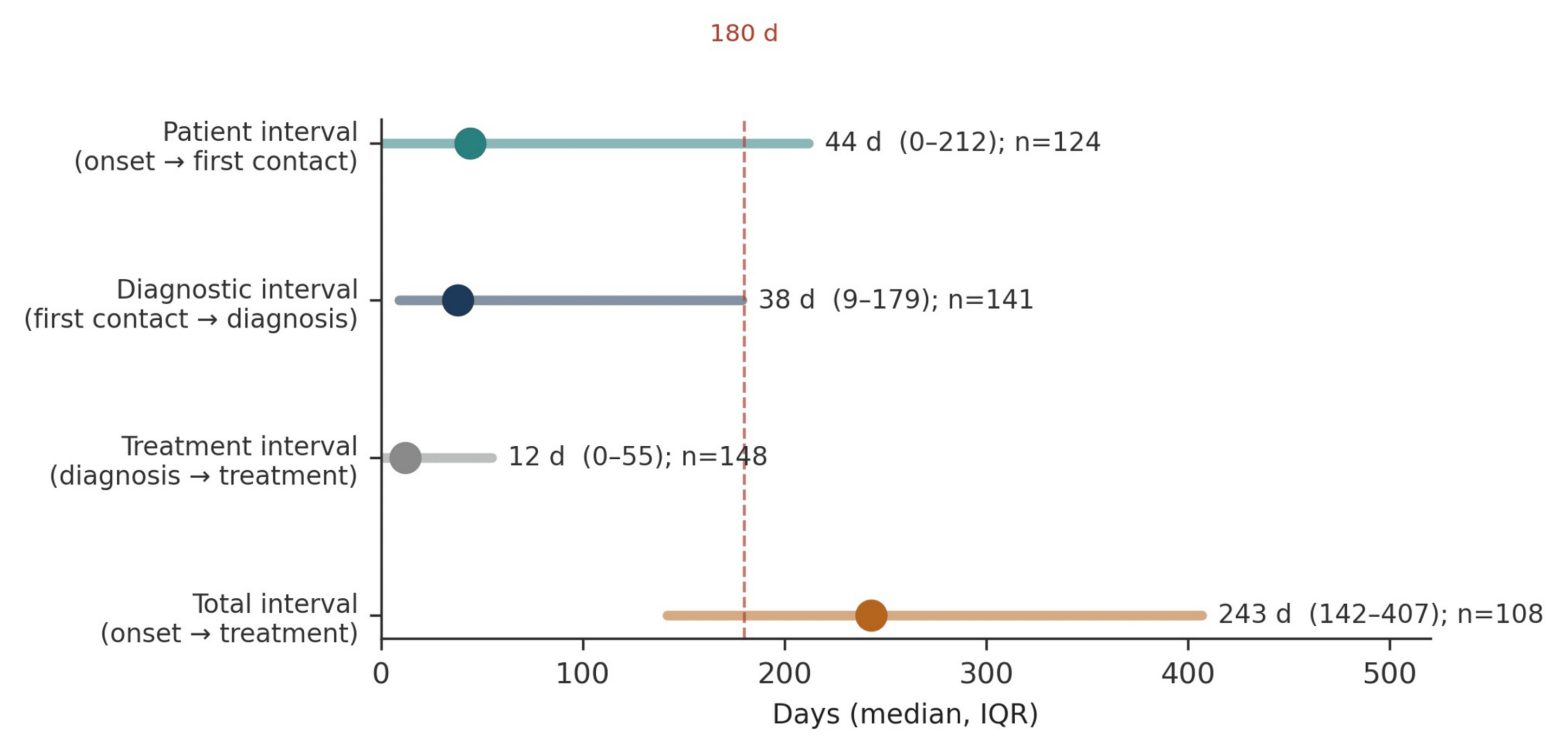
Diagnostic and treatment intervals (Aarhus framework): medians with interquartile range; n per interval shown. The dashed line marks 180 days.

Table 2 and Figure 3 summarize the time elapsed along the diagnostic pathway. The total journey from symptom onset to treatment lasted a median of 243 days (IQR 142-407) - roughly eight months - and exceeded 180 days in 72 of 108 patients (67%). The diagnostic interval, from first medical contact to histopathological confirmation, was longer than 90 days in 51 of 141 patients (36%).

**Table 2:** Diagnostic and treatment intervals (Aarhus framework).

Interval (Aarhus)	Median days (IQR)	n	Prolonged, n (%)
Patient (onset → first contact)	44 (0-212)	124	-
Diagnostic (first contact → diagnosis)	38 (9-179)	141	51 (36%) >90 days
Treatment (diagnosis → treatment)	12 (0-55)	148	-
Total (onset → treatment)	243 (142-407)	108	72 (67%) >180 days

When early- and advanced-stage disease were compared (Table 3), carcinoembryonic antigen (CEA) was markedly higher in advanced disease (median 5.1 vs. 1.3 ng/mL; p < 0.001) and rectal location was more frequent among advanced cases (104/176 (59%) vs. 17/50 (34%); χ²(1) = 9.85, p = 0.002, φ = 0.21). None of the diagnostic intervals differed significantly between groups (all p > 0.33), nor did age, number of symptoms, alarm symptoms (χ²(1) = 1.24, p = 0.27, φ = 0.08), female sex (χ²(1) = 1.74, p = 0.19, φ = 0.09), or prior external misdiagnosis.

**Table 3:** Comparison of advanced- vs. early-stage disease. Continuous variables compared with the Mann-Whitney U test; categorical variables with the χ² test (df = 1; effect size φ): rectal location χ² = 9.85, φ = 0.21; alarm symptoms χ² = 1.24, φ = 0.08; female sex χ² = 1.74, φ = 0.09. CEA, carcinoembryonic antigen; IQR, interquartile range

Variable	Advanced (III-IV)	Early (I-II)	p
Age, years - median (IQR)	41 (35-45)	42 (36-46)	0.442
Patient interval, days - median (IQR)	40 (0-214)	78 (0-200)	0.647
Diagnostic interval, days - median (IQR)	36 (8-181)	43 (15-162)	0.741
Total interval, days - median (IQR)	263 (154-410)	208 (110-398)	0.337
CEA, ng/mL - median (IQR)	5.1 (1.6-24.4)	1.3 (1.0-2.7)	<0.001
Rectal location - n (%)	104 (59%)	17 (34%)	0.002
Alarm symptoms present - n/N (%)	120/152 (79%)	29/41 (71%)	0.27
Female sex - n (%)	80 (45%)	28 (56%)	0.19

**Figure 4:**
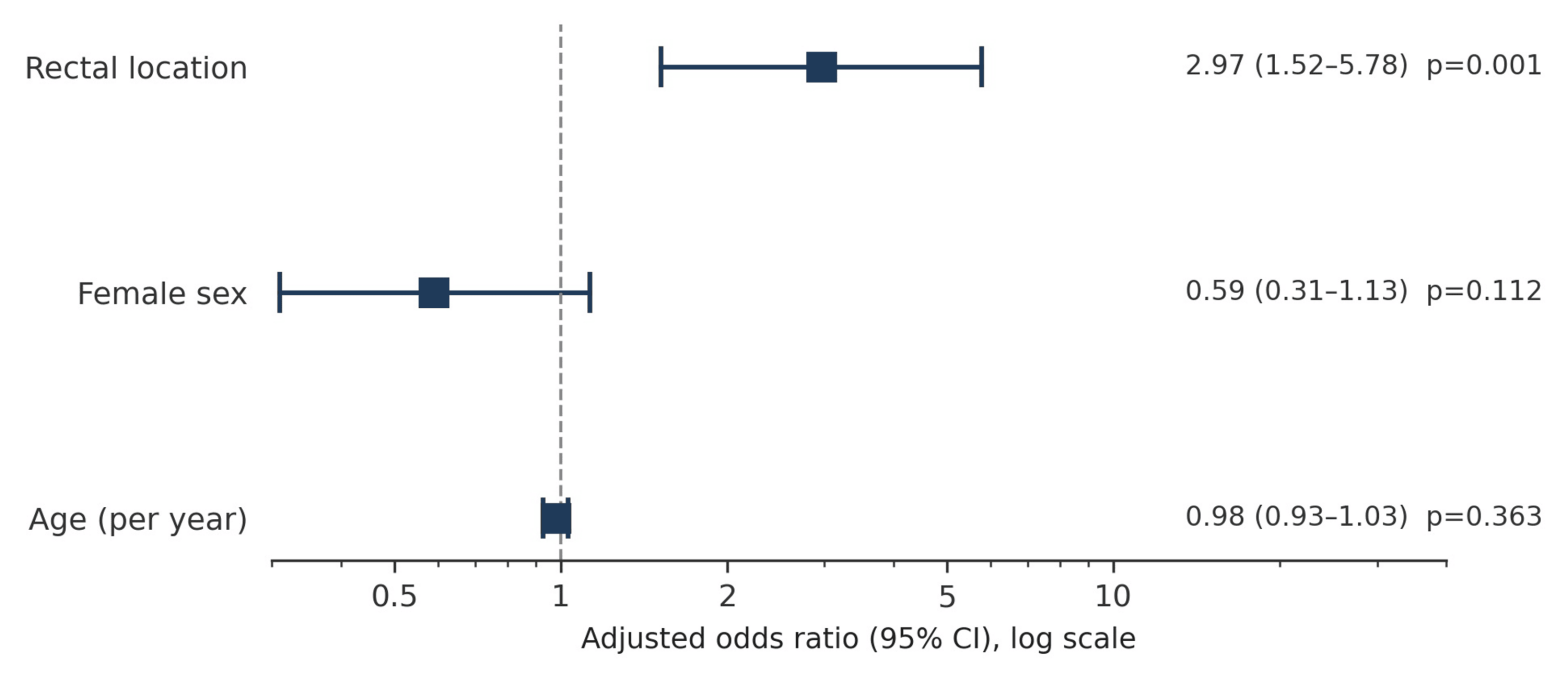
Multivariable logistic regression for advanced-stage presentation: adjusted odds ratios with 95% CI (N = 226). The dashed line marks OR = 1.

In multivariable logistic regression (Table 4, Figure 4; N = 226), rectal location was the only factor independently associated with advanced-stage presentation (adjusted OR 2.97, 95% CI 1.52-5.78; p = 0.001); neither age nor female sex was associated. In a model additionally including a prolonged diagnostic interval (>90 days; n = 141 complete cases), rectal location remained associated (OR 4.31, 95% CI 1.78-10.42; p = 0.001), whereas a prolonged diagnostic interval was not associated with advanced stage (OR 1.11, 95% CI 0.45-2.72; p = 0.824).

**Table 4:** Multivariable logistic regression for advanced-stage presentation (N = 226).

Variable	Adjusted OR	95% CI	p
Rectal location	2.97	1.52-5.78	0.001
Female sex	0.59	0.31-1.13	0.112
Age (per year)	0.98	0.93-1.03	0.363

The findings were confirmed in sensitivity analyses (Table 5). Restricting the cohort to patients diagnosed from 2019 onward (n = 148) preserved the high proportion of advanced stage, the absence of any interval-stage association (all p ≥ 0.56), the higher CEA in advanced disease, and the rectal predominance. Patients with and without a computable interval were comparable in age and stage, and the availability of complete dates reflected record age-concentrated in the 2016-2018 block, rather than any clinical characteristic.

**Table 5:** Characteristics of patients with vs. without computable Aarhus intervals (sensitivity analysis).

Group	n	Median age	Advanced III-IV, n (%)	Rectum, n (%)	Diagnosed ≤2018, n (%)
With computable diagnostic interval	141	41	111 (79%)	80 (57%)	27 (19%)
Without computable diagnostic interval	85	42	65 (76%)	41 (48%)	48 (56%)
With computable total interval	108	41	80 (74%)	57 (53%)	15 (14%)
Without computable total interval	118	41	96 (81%)	64 (54%)	60 (51%)

## Discussion

In this cohort of 226 patients with EOCRC, three findings stand out. First, the disease burden was predominantly advanced: 176/226 (78%) presented at clinical stages III-IV, a proportion higher than typically reported in high-income settings [3] and consistent with prior Mexican series [16,19]. Second, the diagnostic pathway was markedly prolonged, with a median total interval of approximately eight months and two-thirds of patients exceeding six months. Third, rectal location - just over half of patients - was the strongest independent correlate of advanced-stage presentation. These observations extend prior work from our center that compared the clinical profile of early- vs. late-onset disease [16] by quantifying, for the first time in this population, the diagnostic pathway that precedes the advanced presentation.

In EOCRC, the stage at diagnosis appears to depend more on tumor biology than on the time taken to detect it, which would explain why, in our cohort, longer intervals were not accompanied by more advanced disease. These tumors cluster in the distal colon and rectum [4,13] and frequently display mucinous or signet-ring histology and aggressive molecular alterations [12,14-15,20], features that can drive an advanced presentation regardless of diagnostic promptness. The prolonged delay nonetheless demands attention in its own right: a diagnostic pathway of nearly eight months is a shortfall in care that must be corrected [10-11].

CEA was significantly higher in advanced disease, consistent with the predominance of stage IV tumors. The rectal predominance and its association with the advanced stage agree with reports in other young populations [4,13]. The symptom profile-rectal bleeding, tenesmus, and changes in bowel habit-and the prolonged patient interval are consistent with a well-documented feature of EOCRC: because these patients fall below the screening age, they are diagnosed through symptoms that are frequently attributed at first to benign anorectal conditions, delaying access to colonoscopy [9,14].

These findings support pragmatic interventions: a lower threshold for colonoscopy in symptomatic patients younger than 50, structured fast-track referral, and clinician and public awareness that CRC is no longer a disease of older adults [8-9,14] - especially in a country with a high and rising burden of metabolic disease [21-22].

Limitations

This study has limitations inherent to a retrospective, single-center design, whose referral bias concentrates advanced disease and limits generalizability to community settings. Importantly, the approximate recording of symptom onset (non-differential measurement error) and the limited number of computable intervals both bias comparisons toward the null, so our central finding-that delay was not associated with stage-should be interpreted with caution and may partly reflect these constraints. As an observational study, it also cannot exclude residual confounding by unmeasured factors, particularly tumor molecular features, lifestyle, and dietary exposures; in addition, staging was clinical, and comparisons were unadjusted for multiple testing. Prospective, multicenter confirmation is the natural next step.

## Conclusions

In Mexican adults younger than 50, CRC travels nearly eight months from first symptom to treatment and is diagnosed mostly at an advanced stage. That the length of this journey is unrelated to stage is consistent with advanced presentation in young patients reflecting tumor biology more than diagnostic delay, and shifts the target of intervention toward early clinical recognition of the symptomatic patient. Because these findings derive from a single referral center, they should be confirmed in community-based and multicenter cohorts before broader generalization. Characterizing the biology behind this presentation, in prospective cohorts and with molecular study, is the direction these results open for EOCRC in the region.
